# Carboxymethyl kappa-carrageenan polyelectrolyte multilayers: anticoagulant blood-compatible coatings as sustainable alternatives to heparin for blood-contacting surfaces

**DOI:** 10.21203/rs.3.rs-7014360/v1

**Published:** 2025-08-06

**Authors:** Liszt Y. C. Madruga, Somayeh Baghersad, Paulo C.F. da Câmara, Roberta M. Sabino, Matt J. Kipper, Ketul C. Popat

**Affiliations:** a. Department of Chemical and Biological Engineering, Colorado State University, Fort Collins, CO 80526, USA; b. School of Biomedical Engineering, Colorado State University, Fort Collins, CO 80526, USA; c. Laboratory of Petroleum Research, LAPET, Institute of Chemistry, Federal University of Rio Grande do Norte, UFRN, 59078-970 Natal, RN, Brazil; d. Department of Chemical and Biomedical Engineering, University of Wyoming, Laramie, WY, 82071, USA; e. Department of Bioengineering, George Mason University, Fairfax, VA 22030, USA

## Abstract

This research introduces carboxymethyl kappa-carrageenan-chitosan polyelectrolyte multilayers as a promising and sustainable alternative to heparin, used in surface treatments for blood-contacting medical devices. The polysaccharide-based surface coatings have good cytocompatibility and resist microbial adhesion of both Pseudomonas aeruginosa and Staphylococcus aureus. The blood compatibility of surfaces containing carboxymethyl-kappa-carrageenan was directly compared to similar polyelectrolyte multilayers containing heparin. The carboxymethyl-kappa-carrageenan surfaces inhibit whole-blood clotting like heparin-containing surfaces. Blood clotting is mediated by both protein-surface and cell-surface interactions. The carboxymethyl-kappa-carrageenan surfaces adsorb more fibrinogen and less albumin than the heparin surfaces, and they also exhibit reduced platelet and leukocyte adhesion, compared to heparin surfaces. These results suggest that carboxymethyl-kappa-carrageenan may represent a sustainable alternative to heparin as an anticoagulant surface modification.

## Introduction

Polysaccharides sourced from plants, algae, and bacterial fermentation have attracted significant attention in the field of biomaterials for their biomimetic properties, biodegradability, and functional versatility.^1,2^ Sulfated polysaccharides in particular have a diverse repertoire of biological activities, including biomechanical, biochemical, antimicrobial, and tissue-protective modes of action.^3–5^ Heparin is an important suflated polysaccharide, widely used in surface coatings on blood-contacting materials for its anti-coagulant activity. The reliance on animal-derived heparin, particularly from porcine sources, presents significant challenges in terms of safety, ethics, and supply chain reliability. The 2008 heparin contamination incident, which resulted in numerous fatalities due to oversulfated chondroitin sulfate, a common by-product of heparin production, underscored the inherent risks associated with animal-based products.^6^ Also, the outbreak of African swine fever in 2018 highlighted the precariousness of global heparin supplies, further emphasizing the need for alternative anticoagulant biomolecules.^7^

Carrageenans are a family of polysaccharides extracted from red seaweeds and composed of glucopyranose and 3,6-anhydrogalactopyranose monomers. Carrageenans are extensively used in the food and pharmaceutical industries for their gelling, thickening, and stabilizing properties.^8–10^ Among the carrageenan family, kappa-carrageenan (KC)^1^ is distinguished by its single sulfate group per disaccharide unit.^1,10^ KC has been used in regenerative medicine and shows immunomodulation and antitumor activity.^11,12^

Chemical modification of polysaccharides is an important route to enhance, develop, or change properties of these biopolymers. For example, sulfation has been used to introduce sulfate groups to prepare polysaccharides with heparin-mimetic properties. However, sulfation chemistries are chemically harsh and hazardous. KC already contains sulfate groups, so beginning with this as a natural precursor to heparin mimics avoids harsh and hazardous sulfation chemistries.

Carboxymethyl kappa-carrageenan (CMKC), derived from kappa-carrageenan, offers a structurally similar yet more sustainable and safer substitute for heparin.^3^ Unlike traditional heparin, which is susceptible to contamination and supply disruptions, CMKC is plant-derived, reducing dependency on animal sources and mitigating the risks of viral or prion contamination. This innovation addresses critical safety concerns while also promoting ethical and sustainable practices in the development of blood-compatible surfaces for blood-contacting medical surfaces.

Carboxymethylation of polysaccharides is a widely studied chemical modification for development of new biomaterials.^9,13^ The carboxymethylation reaction has been used to modify KC properties leading to the development of CMKC. CMKC has a similar structure to heparin, which is used clinically as an anticoagulant, and to improve the blood compatibility of surfaces; it contains ionizable carboxylic acid and sulfate groups. The introduction of carboxyl groups through carboxymethylation was intended to enhance both the structural and functional similarity of CMKC to heparin. Sulfate groups (-OSO_3_^−^) impart a strong negative charge that enables electrostatic interactions with positively charged proteins such as antithrombin III (ATIII), a key inhibitor of coagulation. Meanwhile, the added ionizable carboxyl groups (-COO^−^) improve hydrophilicity and water retention at the material interface, reducing nonspecific protein adsorption and cellular adhesion. Together, this modification strengthens CMKC’s heparin-like functionality in a more sustainable, plant-derived polymer. Recent reports on the use of CMKC as a biomaterial have focused on the gelation process, its effects on blood clotting and bacterial growth, as well as properties such as cytocompatibility with human cells, antioxidant activity, and antibacterial activity.^3,14^ In a recent study by our research group, CMKC was integrated into poly (vinyl alcohol) nanofibers (PVA-CMKC) to create a novel wound dressing. The PVA-CMKC nanofibers showed improved hemostatic properties and reduced bacterial adhesion and viability, particularly against *Staphylococcus aureus* and *Pseudomonas aeruginosa*, demonstrating their potential as effective wound dressings.^15^ Another recent study demonstrated the use of CMKC in 3D-printed poly-ε-caprolactone scaffolds, enhancing pre-osteoblast proliferation and osteogenic activity. This innovative approach underscores the potential of CMKC in bone tissue engineering.^16^

Surface modification is a critical strategy for enhancing the biocompatibility and functionality of medical implants.^17–19^ Polyelectrolyte multilayers (PEMs), fabricated using the layer-by-layer (LbL) technique, are an excellent surface modification, as they offer exceptional control over nano-scale thickness and composition. The LbL deposition process involves depositing alternating layers of polycations and polyanions onto a charged surface.^20,21^ These PEMs are highly customizable due to their compatibility with a range of biopolymers, facilitating biocompatibility and biodegradability. Their unique properties allow for specific biological interactions, such as controlled cell attachment and antibacterial efficacy. Also, scalability of PEM formation makes this method suitable for development of advanced medical coatings.^22–24^ Chitosan, a widely used polycation in PEM assembly, was selected due to its positive charge, bioactivity, and compatibility with anionic biomolecules such as heparin or CMKC. For example, previous research has investigated the blood compatibility of heparin-chitosan PEMs.^25–28^

**Figure 1.**
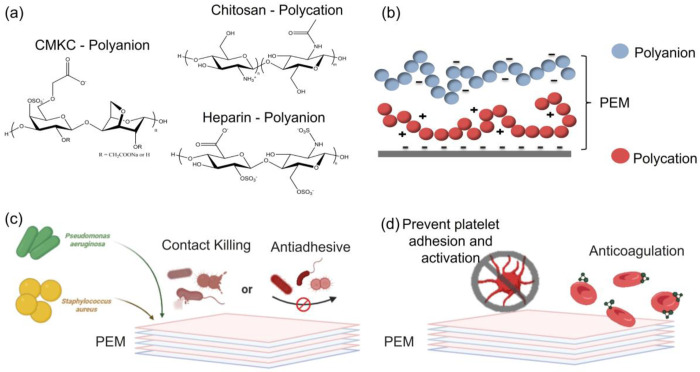
Schematic representation of PEMs and their application mechanisms: chemical structures of polyelectrolytes (a), PEM formation process (b), antibacterial mechanism (c), and hemocompatibility mechanism (d).

Blood-compatibility requires that surfaces (i) be non-toxic to mammalian cells, (ii) inhibit bacterial infection (iii), control plasma protein adsorption, and (iv) prevent blood-clotting. Moreover, surface coatings must be durable to sterilization methods, and stable in biological environments for lengths of time suitable for their intended use. PEMs are electrostatically stabilized and are stable in biological media for days. They can also be chemically crosslinked to improve their stability for biological and medical applications.^29,30^ This work hypothesizes that blood-compatible characteristics would be imparted to PEM surfaces by carboxymethyl kappa-carrageenan. To test this hypothesis, the blood compatibility of PEMs containing CMKC was compared to that of PEMs containing heparin, with the aim of establishing plant-derived CMKC as an alternative to heparin in coatings for blood-contacting materials. In this study, oxidized glass surfaces were modified with PEMs containing CMKC as the polyanion and chitosan as the polycation, to develop hemocompatible and antibacterial CMKC-chitosan (CMKC-CHI) PEMs. The glass surfaces were chosen because they are suitable substrates for all of the outcomes measured in this work. The PEM coating method is versatile; we have translated PEM coatings to polymers, metals, ceramics, nanostructured materials and bone^30–35^. This environmentally friendly process avoids the use of hazardous solvents or waste. The PEMs, with different layer numbers and different terminated layers (chitosan or CMKC), were examined to assess surface coverage and how the final layer alters the surface physical chemistry. These PEMs are cytocompatible toward mammalian cells, are antiadhesive and antimicrobial against *P. aeruginosa* and *S. aureus*, and reduce blood protein adsorption, platelet adhesion/activation, and blood coagulation (Figure 1). Furthermore, the blood compatibility of CMKC-CHI PEMs is superior to similar PEMs containing heparin as the polyanion.

## Materials and Methods

### Materials

Chitosan with a 90% degree of deacetylation and a molecular weight of 8.7 × 10^4^ g/mol was sourced from MP Biomedicals, USA (CAT NO 150597). The chitosan was purified by dissolving in a 1.0% w/v solution in 1.0% v/v aqueous acetic acid, followed by dialysis against deionized water using a 7,000 MWCO membrane for two days, refreshing the water twice daily. Sodium kappa-carrageenan (KC) with a molecular weight of 3.9 × 10^5^ g/mol (C10103, Sigma-Aldrich) and monochloroacetic acid were used without further purification. Heparin, a sulfated glycosaminoglycan with 12.5% sulfur content and a molecular weight of 14.7 kDa, was procured from Celsus Laboratories, Cincinnati, OH, USA (LOT NO PH-59707@176). Sodium acetate, glacial acetic acid, and ethanol (>98 vol%) were purchased from Fisher Scientific, Waltham, MA, USA, and Acros Organics, Morris Plains, NJ, USA, respectively. 11-Mercaptoundecanoic acid (95%) was obtained from Sigma-Aldrich. Deionized water (DI water) with a resistivity of 18.2 MΩ cm was produced using a Millipore water purification system. Adipose-derived stem cells (ADSC) were isolated by Prof. Kimberly Cox-York from the Department of Food Science and Human Nutrition at Colorado State University (CSU) from a previous study involving abdominal and femoral subcutaneous adipose tissue biopsies; the protocol was approved by the CSU Institutional Review Board. Phosphate-buffered saline solution (PBS) without calcium and magnesium was acquired from Gibco, USA.

### Carboxymethylation of kappa-carrageenan

Carboxymethyl-kappa-carrageenan was synthesized using Williamson’s ether synthesis technique. Initially, 10 g of kappa-carrageenan was dissolved in a solution of 80% w/v 2-propanol (200 mL). After that, sodium hydroxide solution (20% w/v, 20 mL) was added gradually over a period of 15 minutes. The mixture was vigorously stirred at 40 °C for one hour. Following this, monochloroacetic acid (8.75 g dissolved in NaOH solution) was added slowly while the temperature was increased to and held at 55 °C for four hours. This is an MCA:KC monomer ratio of 3:5. In previous work, we showed that this procedure results in CMKC with a degree of substitution of 1.1 ^36^. After the reaction, the substance underwent vacuum filtration and washing with both an 80% ethanol/water solution followed by pure ethanol. The filtered solid was then dissolved in deionized water overnight before undergoing dialysis against water until its conductivity fell below 20 mS·cm^−1^ for purification purposes. Finally, the material was freeze-dried to produce CMKC with a substitution degree of 1.1. This product has been characterized previously by ^1^H NMR, ^13^C NMR, and FTIR.^3^

### Polyelectrolyte multilayer preparation

PEMs were prepared using chitosan as the polyanion and either heparin or CMKC as the polyanion (Figure 1a), using procedures described elsewhere.^37^ Detailed procedures are provided in the supporting information (§S1.1).

### Characterization

Fourier transform surface plasmon resonance was used to monitor the LbL assembly of PEMs in situ. The resulting PEMs were evaluated by X-ray photoelectron spectroscopy, contact angle goniometry, and atomic force microscopy, as described in §S1.2.

### Cytocompatibility studies

The experimental methodologies, including cell culture, viability, and cytotoxicity assessments, as well as evaluations of adhesion and proliferation on PEM-coated surfaces, are comprehensively detailed in §S1.3 of the supporting information. Cytocompatibility of PEMs is compared tissue-culture polystyrene TCPS (non-cytotoxic control). The adipose-derived stem cells (ADSCs) used in this work are cultured on TCPS. A media containing 1% Triton-X, which ruptures the cell membranes was used as the 100% cytotoxic control.

### Antibacterial activity studies

The methods of the antibacterial activity assays, aimed at evaluating the effectiveness of PEM-coated surfaces against *S. aureus* (gram-positive) and *P. aeruginosa* (gram-negative), are detailed in §S1.4 of the supporting information.

### Hemocompatibility studies

For evaluating blood interactions with PEM surfaces, the protocol began with the sterilization of all samples, including control surfaces of glass and tissue culture polystyrene (TCPS). This was achieved by immersing the surfaces in 70% ethanol for 30 minutes, followed by rinsing three times with DI water. PEMs with 16 layers were evaluated; the number of layers was selected based on preliminary experiments indicating optimal results with CMKC as the terminating layer. Additionally, heparin-chitosan (HEP-CHI) 16-layered surfaces were included for comparison, given known blood compatibility of heparin.

#### Protein adsorption on PEM surfaces.

Two important blood proteins, fibrinogen and albumin were selected for analysis. The sterilized substrates were incubated in a 24-well plate with a 100 μg/mL solution of fibrinogen and albumin in PBS. The incubation was conducted at 37 °C for 2 hours on a horizontal shaker set at 100 rpm in a 5% CO2 atmosphere. After the incubation period, the protein solution was aspirated, and the surfaces were rinsed twice with PBS and once with deionized water to remove non-adherent proteins. The surface composition of the adsorbed proteins was then characterized using XPS, focusing on the nitrogen increment due to adsorbed proteins on the elemental composition obtained from the high-resolution C1s, O1s, N1s and S2p XPS spectra.

#### Platelet adhesion and activation.

Blood was collected from two healthy donors, who signed formal consents for this study, using venous phlebotomy. All practices were approved by the Colorado State University Institutional Review Board, which agrees with the National Institutes of Health’s “Guiding Principles for Ethical Research.” The blood was drawn into 10-mL EDTA-coated vacuum tubes and centrifuged at 100 × g for 15 minutes to separate plasma containing platelets and leukocytes from red blood cells. The platelet-rich plasma (PRP) was then allowed to rest for 10 minutes before further use.^38^

Platelet adhesion was investigated using fluorescence microscopy. Sterilized PEM samples were placed into a 24-well plate and incubated with 200 μL of PRP on a horizontal shaker at 100 rpm, 37 °C, and 5% CO2 for two hours. After incubation, non-adherent platelets were removed by rinsing with PBS. Then the same staining procedure detailed in §S1.4 of the supporting information was followed. Imaging was performed using a Zeiss Axiovision fluorescence microscope. Three non-overlapping images from each of three replicate samples per condition were analysed using ImageJ Software to estimate the percentage of the surface covered by adhered platelets.

Platelet activation was characterized using SEM. After incubation with PRP as described above, the un-adhered platelets were removed, and the surfaces were rinsed twice with PBS. The adhered platelets were fixed and imaged following the same procedure in section §S1.4 of the supporting information. The SEM images were used to visualize platelet morphology and assess platelet activation in response to the PEM surfaces.

#### Whole blood clotting.

Human blood was collected from healthy donors into 3-mL vacuum tubes without any anticoagulants. The collection process was conducted by a trained phlebotomist to ensure proper and ethical blood withdrawal. The sterilized PEM samples were arranged on a 24-well plate. To initiate the clotting process, a 7.0-μL drop of whole blood was placed on each of the samples. The clotting was allowed to proceed for specified time intervals of 15 and 30 minutes to observe different stages of the clotting process. After each clotting period, 500 μL of DI water was added to the surfaces. These were then gently agitated for 5 minutes on a horizontal shaker. This agitation was aimed at lysing the red blood cells and dissolving any un-clotted blood, thereby releasing free hemoglobin into the DI water. The absorbance of the free hemoglobin was subsequently measured using a plate reader (Molecular Devices Spectra Max M3) at a wavelength of 540 nm. A control sample for 100% free hemoglobin release was obtained by solubilizing a blood sample in water and measuring its absorbance immediately after collection (0 min).

### Statistical analysis

For each type of PEM, a minimum of three distinct samples were utilized in all experiments. The data are reported as the mean ± standard deviation. To identify significant differences between groups, a one-way analysis of variance (ANOVA) was conducted at a significance level of p = 0.05, followed by Tukey’s honest significant difference test for post-hoc analysis.

## Results and Discussion

### Characterization of PEMs

**Figure 2.**
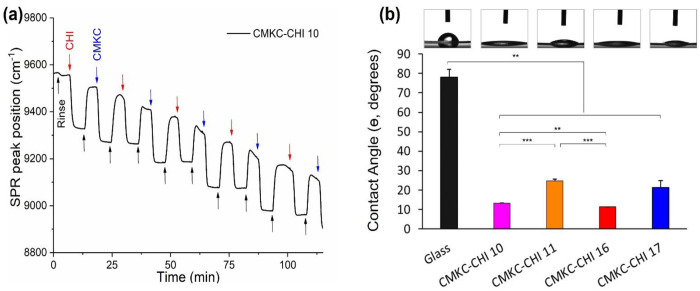
Kinetics of 10-layer PEM assembly of CMKC-CHI 10 monitored by in situ FT-SPR (a) and static water contact angles for CMKC-CHI PEM surfaces with different number of layers (b). Values represent mean ± standard deviation (*n* = 3). Glass was considered as a control; *** indicates *p* ≤ 0.001, ** indicates *p* ≤ 0.01.

The successful LbL assembly of CMKC-CHI PEMs is confirmed using in situ Fourier-Transform Surface Plasmon Resonance (FT-SPR) (Figure 2a).^39^ The decrease in the FT-SPR peak position during the rinse step following each adsorption step confirms consistent LbL growth of the PEM with precise control over the PEM thickness by changing the number of layers.^40^ This decline in the FT-SPR peak position is attributed to the irreversible adsorption of charged polyelectrolytes onto the surface, along with differences in refractive index between rinse and polymer solutions. Our construction of 10-layer PEMs highlights the potential for developing advanced nanoscale coatings.

The XPS survey spectra (Figure S1a and S1c) show the characteristic sulfur (S2p) and nitrogen (N1s) peaks, indicating that the sulfate groups from CMKC or heparin and amine groups from chitosan have been successfully incorporated into the PEMs. These elements are consistently present in all samples, even those with different terminal layers, which confirms that the deposited layers are very thin.

Details of the carbon bonding environment in the PEMs are revealed through the high-resolution C1s XPS spectra (Figure S1b). By deconvolving these spectra into their component peaks, specific functional groups can be identified. The relative intensities of the -COOH and O=C–N peaks vary depending on whether the terminal layer of the PEM is the polyanion (CMKC) or the polycation (chitosan); Polyanion-terminated (10- and 16-layer) PEMs exhibit a more prominent -COOH peak, suggesting a higher concentration of carboxylic acid groups near the surface, whereas chitosan-terminated PEMs have a greater amide peak contribution.^41^ Similarly, the high resolution N1s and S2p spectrum (Figure S2) show higher intensity of amide group in the N1s spectra for the polycationic terminated layers and a higher sulfate peak in the S2p spectra for the anionic terminated layers. In addition, atomic percentage of the PEMs obtained through the high-resolution spectra confirmed the construction of layers by the increment of sulfur on CMKC terminated layers and increment of nitrogen on chitosan terminated layers (Table S1). These differences in surface chemistry could impact the bioactivity of the PEMs.

Comparison of the survey and high-resolution C1s spectra for CMKC-CHI and HEP-CHI PEMs in Figure S1 indicates a structural similarity between CMKC and heparin, particularly in the arrangement of carboxylic acid and sulfate groups. This similarity suggests that CMKC could be a prospective alternative to heparin in the preparation of PEM surface coatings. CMKC may also mimic the biofunctional characteristics of heparin, which is extensively used in diverse biomedical applications due to its anticoagulant and biocompatible properties.^42^

The contact angle measurements (Figure 2b) indicate that all the PEMs significantly enhance the surface wettability when applied to glass. The untreated glass has a high contact angle, suggesting that it is relatively hydrophobic. The PEMs have much lower water contact angles than glass, indicating that the PEMs present a more hydrophilic surface.^43^ This decrease is caused by hydrophilic functional groups in the polysaccharides.

The polycation-terminated (chitosan-terminated) PEMs with odd layer numbers have higher water contact angles than the polyanion-terminated PEMs with even layer numbers. Chitosan is relatively hydrophobic compared to the polyanions, and terminating a PEM with chitosan may result in complexation between amines in chitosan and negatively charged functional groups in the heparin or CMKC.^44–46^

**Figure 3.**
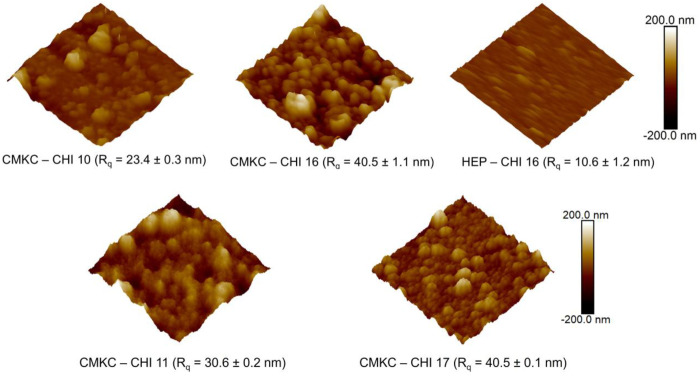
Representative 2.0 μm × 2.0 μm AFM topographic images of the PEMs taken in PBS.

Surface roughness, as determined using AFM, is an important factor that affects the interaction of materials with biological systems.^47,48^ The 10- and 16-layer PEMs were selected based on previous studies from our group that demonstrated optimal nanoscale roughness and PEM assembly behavior at these layer numbers. These configurations provided sufficient coating

thickness to mask the underlying glass substrate while maintaining a nanometric roughness. In Figure 3, the AFM images show different roughness values for the surfaces examined: CMKC-CHI 10, CMKC-CHI 11, CMKC-CHI 16, CMKC-CHI 17 and HEP-CHI 16, with root-mean-square roughness measurements of approximately 23.4 nm ±0.3 nm, 30.6 nm ±0.2 nm, 40.5 nm ±1.1 nm, 40.5 nm ±0.1 nm and 10.6 nm ±1.2 respectively. Alternating the terminated layer of CMKC to chitosan from 10 to 11 layers changes the roughness, probably due to the higher molecular weight of CMKC, and the conformation of chitosan when adhering to the surface. However, for higher number of layers there was no difference between the roughness, suggesting that the surface is near saturation. Additionally, all CMKC PEMs presented higher roughness than heparin PEMs. These surface variations in nanotopography are known to have an impact on cellular interactions by influencing protein conformation, orientation, and activity upon adsorption which ultimately affects cell adhesion, proliferation, and differentiation.^49^

In summary, the XPS analysis indicates that CMKC-CHI PEMs have been successfully fabricated with clear evidence of each polymer’s presence and the potential to customize the surface chemistry through the terminal layer. The water contact angle measurements highlight the connection between surface chemistry, terminating layer of the PEM, and wettability and help to confirm the successful modification of the surfaces. Complementing this, AFM analysis has provided essential insights into the topographical characteristics, revealing a controllable roughness that may have significant implications for subsequent protein surface interactions and biological responses to surfaces. This study sets the foundation for further investigation of important biological responses to CMKC-containing PEMs.

### Cell viability and toxicity assays

Viability and cytotoxicity assays carried out on CMKC-CHI PEMs demonstrated a consistent level of cytocompatibility across different numbers of layers (Figure S3). The cytotoxicity of PEMs was assessed using the LDH enzyme reaction method to determine the proportion of cells that would be damaged or die upon contact with the surfaces.^50^ When cells experience damage to their plasma membrane or are lysed, the LDH enzyme is released into the extracellular medium and can be quantified by reacting with a tetrazolium salt. This results in a red product that can be measured by absorbance at 490 nm.^51^ Figure S3a depicts the percentage cytotoxicity findings for CMKC-CHI PEMs tested with varying numbers of layers. The material cytotoxicity percentage is expressed relative to lysed cells, representing 100% cytotoxicity. For a material to be considered cytotoxic, it must have a value for cytocompatibility below 90%, i.e., cytotoxicity percentage values greater than 10% as per ISO 10993-5:2009 standard.^14^ As shown in Figure S3a, the cytotoxicity percentages for the different CMKC-CHI PEMs ranged between approximately 5% and 8%, all of which are below the 10% cytotoxicity threshold. Therefore, none of the samples demonstrate any evidence of cytotoxic activity since all values were below this threshold. None of the samples exhibit cytotoxicity that is significantly different from the control (TCPS).

The cell viability assay uses Alamar Blue reduction as a measure of cellular metabolic activity (Figure S3b). Higher reduction percentages are associated with increased cell viability.^52^ In this context, the reduction of Alamar Blue ranged from approximately 55% to 75% across the CMKC-CHI PEMs. All samples displayed no significant differences in viability compared to the control, suggesting that all tested PEMs support cell survival.

The combined findings from the viability and cytotoxicity assessments suggest that the CMKC-CHI PEMs exhibit cytocompatibility regardless of their number of layers. The uniform cell viability observed across all specimens implies that the chemical composition and surface attributes of the PEMs support cell metabolic function. Moreover, the absence of significant cytotoxic effects indicates that these multilayers do not cause membrane impairment or cell disintegration, providing additional evidence of their compatibility with biological systems.

### Adhesion and proliferation assays

**Figure 4.**
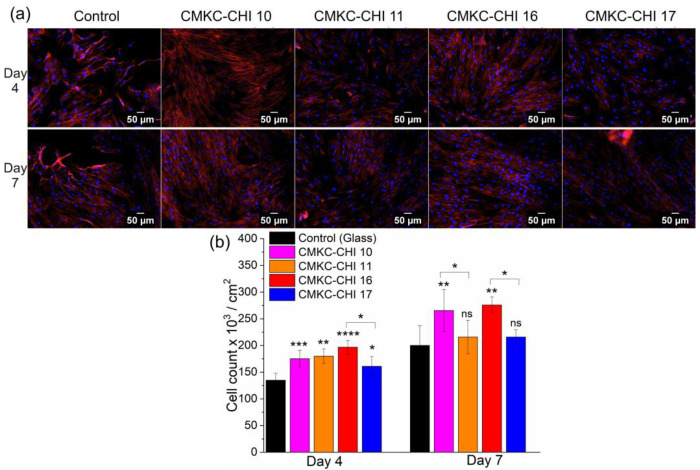
Fluorescent images of ADSCs taken at 10× magnification on PEM surfaces. Red is rhodamine phalloidin for the cell cytoskeleton, and blue is DAPI for the nuclei (a). Cells per area (b). All images and data were taken after 4 and 7 days of culture. Scale bars on fluorescence microscopy images represent 50 μm. Values represent mean ± standard deviation (*n* = 3). ****p ≤ 0.0001, ***p ≤ 0.001, **p ≤ 0.01, *p ≤ 0.05, and “ns” *p* ≥ 0.05, compared to glass.

Adhesion and proliferation of ADSCs on CMKC-CHI PEMs were assessed, revealing that cell spreading was more limited on control samples compared to the extensive distribution observed on the PEMs (Figure 4a).^53^ Figure 4b demonstrates that on day 4, more cells adhered to all CMKC-CHI PEMs compared to the untreated glass used as a control. On day 4, the 16-layer (CMKC-terminated) PEM showed a 28% increase in cell number compared to the 17-layer (chitosan-terminated) PEM; by day 7, both the 10- and 16-layer (CMKC-terminated) PEMs exhibited a 32% and 29% increase, respectively in cell numbers compared to the 11- and 17-layer (chitosan-terminated) PEMs, suggesting the CMKC as terminating layer improves cell attachment. In contrast, the chitosan-terminated layers do not have statistically significantly higher cell numbers relative to the glass control on day 7. This indicates that the terminal layer of these PEMs can influence cell proliferation.

### Antibacterial activity

Based on the results from the mammalian cell viability, adhesion and proliferation experiments, CMKC-terminated layers were selected for further antimicrobial activity assays. The antibacterial characteristics of CMKC-CHI 10 and CMKC-CHI 16 PEMs were investigated against two prevalent and medically important bacteria: *S. aureus* and *P. aeruginosa*.^54^

The antibacterial activity of CMKC-CHI 10 and CMKC-CHI 16 PEM coatings showed significant growth inhibition of *P. aeruginosa* and *S. aureus* (Figure S4) compared to the glass surface. For *P. aeruginosa*, both coatings effectively inhibited growth. In the case of *S. aureus*, growth inhibition was less pronounced, but the trend of increased effectiveness over time was still evident, with the 16-layer coating providing slightly better results. Since the PEMs do not release anything to solution, a high antibacterial activity in solution is not expected.

**Figure 5.**
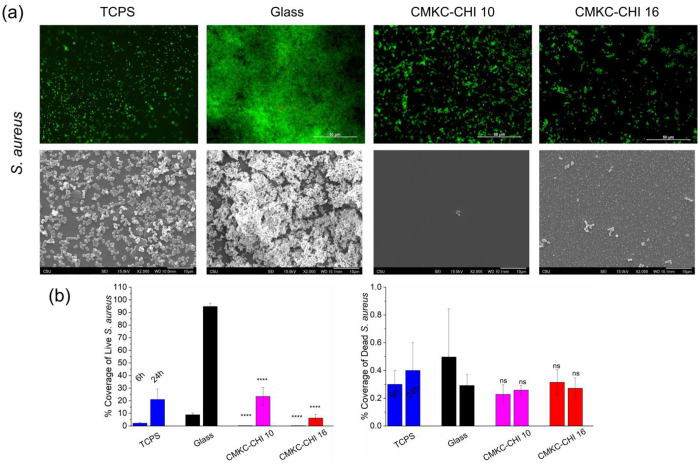
Fluorescence microscopy and SEM images of *S. aureus* (a) on TCPS, glass controls and PEMs after 24 h. Live bacteria are represented in green (SYTO 9 stain) and dead bacteria in red (propidium iodide stain). Original magnification of the SEM images is 2000×. Scale bars on fluorescence microscopy and SEM images represent 50 and 10 μm, respectively. Percentage of coverage for live and dead *S. aureus* adhered to the surfaces after 6 and 24 hr (b). Glass was considered as control. **** *p* ≤ 0.0001 and “ns” *p* ≥ 0.05 compared to glass control at same time point.

**Figure 6.**
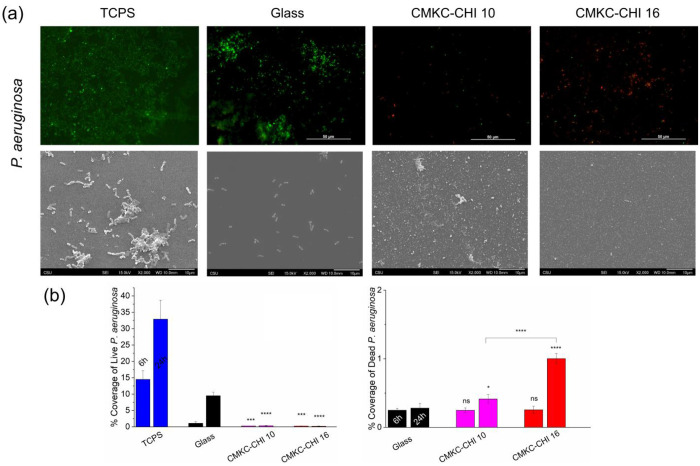
Fluorescence microscopy and SEM images of *P. aeruginosa* (a) on TCPS, glass controls and PEMs after 24 h. Live bacteria are represented in green (SYTO 9 stain) and dead bacteria in red (propidium iodide stain). Original magnification of the SEM images is 2000×. Scale bars on fluorescence microscopy and SEM images represent 50 and 10 μm, respectively. Percentage of coverage for live and dead *P. aeruginosa* adhered to the surfaces after 6 and 24 hr (b). Glass was considered as control. **** *p* ≤ 0.0001, *** *p* ≤ 0.001, ** *p* ≤ 0.01, * *p* ≤ 0.05, and “ns” *p* ≥ 0.05 compared to glass control at same time point.

Coating glass with 10-layer or 16-layer CMKC-CHI PEMs dramatically reduces the attachment of both live *S. aureus* (a Gram-positive bacterium) and live *P. aeruginosa* (a Gram-negative bacterium), at both 6 h and 24 h time points. Both types of bacteria are capable of rapid biofilm formation on surfaces that support their attachment. Representative fluorescence microscopy and scanning electron microscopy images of bacteria on glass controls and both PEMs for the 24 h time point is shown in Figures 5a (*S. aureus*) and 6a (*P. aeruginosa*). The corresponding images at the six-hour time point are shown in the supporting information (Figure S5). The percentage area covered by both live bacteria and dead bacteria from the fluorescence micrographs is quantified for each surface type and bacteria in Figures 5b and 6b. For both bacteria, increasing the number of layers further decreases live bacterial attachment, indicating that thicker PEMs have greater antiadhesive activity. For instance, CMKC-CHI 16 reduces the live *S. aureus* coverage by around 85% compared to the glass control after 24 h, while the 10-layer PEM reduces it by approximately 65-70%, demonstrating the more effectiveness of the thicker PEM. In the case of *P. aeruginosa*, both the 10-layer and 16-layer PEMs reduce live bacterial coverage to less than 0.5%, indicating that even the thinner PEM is highly effective at preventing bacterial attachment, with the thicker PEM providing comparable results. Moreover, SEM analysis (Figure 5a and 6a) shows some evidence of morphological damage to bacteria on the PEM-coated surfaces, and no biofilm formation was observed on PEM-coated surfaces.

While the PEM-coatings are antiadhesive towards Gram-positive *S. aureus*, the PEMs also have additional bactericidal activity toward *P. aeruginosa*. Figure 5b shows that on uncoated glass, the percentage coverage of the surface with live bacteria increases between 6 to 24 hours. In contrast, both CMKC-CHI 10 and CMKC-CHI 16 not only inhibit the growth of *P. aeruginosa* but also significantly increase the percentage of dead bacteria. The higher number of dead bacterial cells on the CMKC-CHI 16 surfaces after 24 hours suggests a more potent antimicrobial action than CMKC-CHI 10.

Chitosan possesses inherent antibacterial properties due to its cationic nature, which disrupts bacterial membranes by interacting with negatively charged cell walls. The observed effects likely result from a synergistic interaction between CMKC and chitosan. PEMs containing CMKC have various characteristics that could confer antibacterial properties. The increased hydrophilicity resulting from CMKC might facilitate the formation of a hydration layer, acting as a barrier against bacterial colonization.^55^ Additionally, the presence of anionic groups like carboxylate and sulfate in CMKC has the potential to disrupt bacterial membranes and interfere with essential cellular functions, leading to bacterial death.^56^ CMKC has been shown to inhibit the growth of both Gram-positive and Gram-negative bacteria. The acidic environment created by the sulfate and carboxylate groups, coupled with the heightened nucleophilicity of the polymer, may account for observed antibacterial activity.^57,58^ This suggests a potentially greater impact on Gram-negative bacteria due to their more permeable lipopolysaccharide membrane; however, results also indicate substantial efficacy against *S. aureus* as well as *P. aeruginosa* bacteria.^59–61^

### Hemocompatibility studies

#### Protein adhesion.

Based on the superior activity of the CMKC-CHI 16 PEMs in antimicrobial activity assays, this condition was compared to HEP-CHI 16 PEMs for all hemocompatibility studies. Heparin is widely used as an anticoagulant agent for inhibiting thrombin activation on surfaces of blood contacting materials.

Thrombus formation is a complex physiological process that involves the sequential cellular (activation and aggregation of platelets), and protein (coagulation cascade culminating in the conversion of fibrinogen into a fibrous fibrin network) processes to form a blood clot.^62^ The initial layer of proteins that adheres to biomaterial surfaces plays a critical role in influencing subsequent protein interactions and biological processes during clot formation. Albumin, which is abundant in blood, may serve as either an inhibitor or promoter of coagulation depending on its conformation after adhering.^63^ Fibrinogen, with its spindle-shaped structure, is essential for clot formation as it transforms into polymerizable fibrin during the coagulation cascade. Adsorbed fibrinogen can also provide binding sites for platelets, thereby stimulating platelet adhesion. This intricate interaction between protein adhesion and conformational changes significantly impacts the potential thrombotic risk associated with biomaterials used in blood-contacting medical devices.^64–67^ Therefore, we investigate protein adsorption, platelet adhesion and activation, and whole blood clotting kinetics to characterize blood-surface interactions.

In this study, XPS was used to examine the adsorption characteristics of human albumin and fibrinogen on PEM surfaces (CMKC-CHI 16 and HEP-CHI 16). Albumin has the potential to either hinder or promote clot formation depending on its configuration, while fibrinogen increases platelet adhesion and activation by exposing binding sites for platelets.^68^ High-resolution spectra were obtained for these surfaces before and after albumin and fibrinogen adsorption (Figure S6 in the supporting information and Table 1). The XPS survey analysis indicates that both HEP-CHI 16 and CMKC-CHI 16 PEMs bind fibrinogen and albumin, revealing changes in the corresponding to O, N, C, and S bonding environments in the XPS spectra following protein exposure. The high-resolution C1s spectra include four sub-peaks: aliphatic carbon (C-C), amine (C-N), amide (N–C=O), and carboxyl (COOH).

The XPS-derived nitrogen content in HEP-CHI 16 and CMKC-CHI 16 PEMs indicates differential protein adsorption profiles. HEP-CHI 16 surfaces exhibit a marked preference for albumin over fibrinogen, with nitrogen content increasing from 3.83% to 10.31% post-albumin adsorption. CMKC-CHI 16 surfaces, on the other hand, display a significant fibrinogen affinity, with a nitrogen content rise from 2.33% to 9.55%. This substantial adsorption of fibrinogen may suggest an enhanced surface for initial thrombus formation; however, tight fibrinogen binding may also inhibit their assembly in fibrin fibers.^43^ Surface adsorbed fibrinogen can adopt different structures that impact other phenomena related to blood coagulation^69^. For example, studies of the structure of surface-adsorbed fibrinogen have revealed that the flexible αC region of the protein is involved in fibrin fiber formation^70^, Changes in the structure of adsorbed fibrinogen are also correlated with dynamic changes in platelet adhesion^71^. These factors collectively contribute to the complexity of interactions at the biomaterial-blood interface.

Platelet adhesion and activation. Thrombogenic potential of PEMs were investigated by examining platelet adhesion via fluorescence microscopy and activation using scanning electron microscopy. Platelet activation is associated with rapid cytoskeletal rearrangement that is readily characterized by electron microscopy^72–74^. Unactivated platelets adopt a round morphology; partially activated platelets have short dendrites; moderately activated platelets have long dendritic extensions (longer than the cell body) and pseuodopodia; fully activated platelets are fully spread or adopt a “fried egg” morphology. The performance of CMKC-CHI PEMs and HEP-CHI PEMs was compared to traditional tissue-culture polystyrene and glass controls.

**Figure 7.**
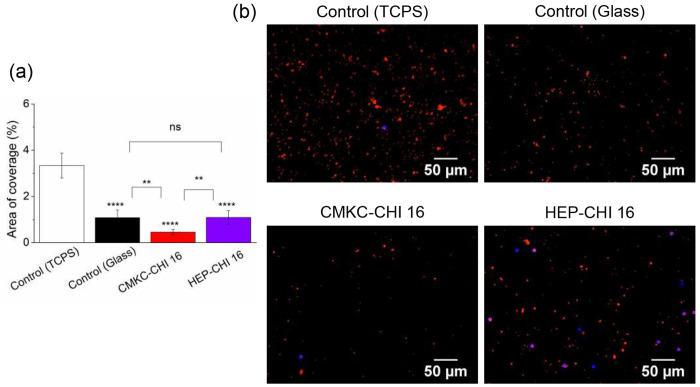
Surface coverage analysis of adhered platelets on control and PEM surfaces (a); Representative fluorescence microscopy images depicting adhered platelets and leukocytes, with platelets stained red using rhodamine phalloidin and leukocytes highlighted in blue with DAPI staining. Scale bars on fluorescence microscopy images represent 50 μm (b). **** *p*≤ 0.0001, ** *p* ≤ 0.01, and “ns” *p* ≥ 0.05 compared to TCPS control at same time point.

Fluorescence imaging showed that TCPS had the highest platelet adhesion, indicating its procoagulant nature. The CMKC-CHI PEMs demonstrated a significant decrease in platelet adhesion, representing around 86% reduction compared to the TCPS. This reduction suggests possible anti-thrombotic characteristics, which could be advantageous for biomedical uses involving clot formation concerns. In contrast, HEP-CHI surfaces exhibited platelet coverage close to the glass control, with a 73% lower coverage compared to TCPS but still higher than CMKC-CHI, suggesting limited antiplatelet effectiveness compared to CMKC-CHI (Figure 7a and 7b). AFM analysis shows that CMKC-CHI PEMs have a rougher surface than HEP-CHI PEMs (Figure 3), indicating a higher potential for fibrinogen adsorption due to increased surface area. However, the interplay between the surface roughness and the high negative charge density of CMKC results in a less favorable fibrinogen conformation for platelet adhesion and activation ^75–77^.

SEM images show examples partially, moderately, and fully activated platelets on the TCPS and glass controls, with most platelets exhibiting some degree of activation. Both the HEP-CHI and CMKC-CHI PEM coatings have very few platelets adhered, which are mostly inactivated.

**Figure 8.**
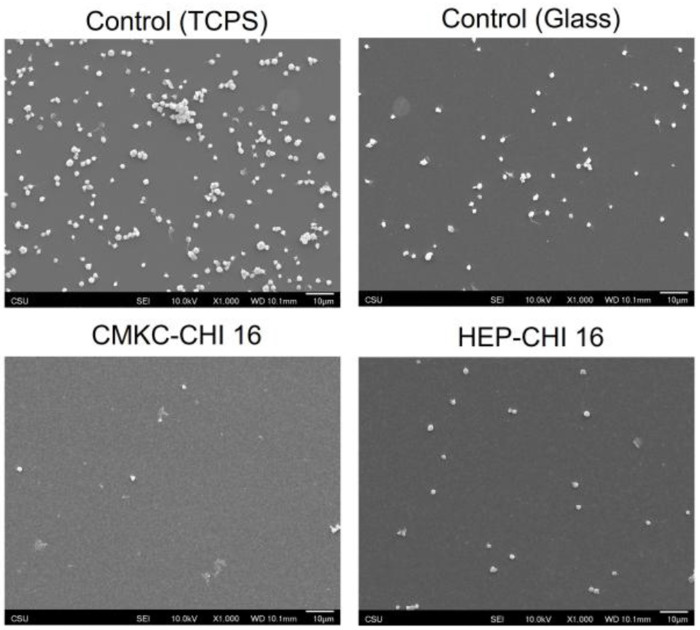
Representative SEM images of adhered platelets on different control and PEM surfaces after 2 hr of incubation in PRP. Scale bars on SEM images represent 10 μm.

Fluorescence microscopy also indicated a higher number of white blood cells on the HEP-CHI PEMs, as evidenced by the blue staining, possibly indicating an immune response (Figure S7 in the supporting information and Figure 7b). The presence of additional white blood cells in SEM images (Figure 8) on the HEP-CHI PEM compared to CMKC-CHI PEM confirms this observation.

The adhesion and activation of platelets play a crucial role in initiating the blood clotting process, ultimately leading to thrombus formation. Hence, creating biomaterial surfaces that can reduce these interactions is extremely important for advancing the field of blood-contacting medical devices.

The CMKC-CHI PEM showed a significant difference compared to the control conditions in terms of platelet adhesion and activation. This decrease in platelet adhesion may be due to the CMKC-CHI PEM showed a significant difference compared to the control conditions in terms of platelet adhesion and activation. This decrease in platelet adhesion may be due to the nature of the surface, which could affect how plasma proteins like fibrinogen are adsorbed and organized. While the CMKC-CHI PEM has higher fibrinogen adsorption, this PEM reduces platelet adhesion possibly by binding fibrinogen tightly in a conformation that is not favorable for platelets to adhere and become active. The topography and chemical composition of a surface have a significant impact on protein adsorption, which in turn affects how platelets interact. Our previous research showed that polyvinyl alcohol nanofibers containing CMKC led to increased adhesion and activation of platelets.^15^ However, our current findings show that the CMKC-CHI PEM surface results in reduced platelet adhesion and activation. This suggests that the topography is a major component for blood compatibility with a surface. PEMs have a roughness of about 40 nm and a 2D structure, resulting in a relatively smaller surface area compared to nanofibers. On the other hand, nanofibers have a rougher surface with an average diameter of 150 nm and a 3D structure, significantly increasing their available surface area for biological interaction. This enhanced topography not only promotes greater protein binding and platelet adhesion but also mimics natural tissue cues, potentially enhancing coagulation responses. From a chemical perspective, PEMs contain numerous carboxyl groups that carry strong negative charges, likely repelling similarly charged platelets and offering potential anticoagulant advantages over nanofibers which have fewer available ionic groups due to crosslinking with polyvinyl alcohol. The nanotopography and chemical properties of the CMKC-CHI PEMs likely create an environment unfavorable for proper conformation of plasma proteins such as fibrinogen, which is essential for platelet interaction and coagulation cascade.

### Whole blood clotting.

**Figure 9.**
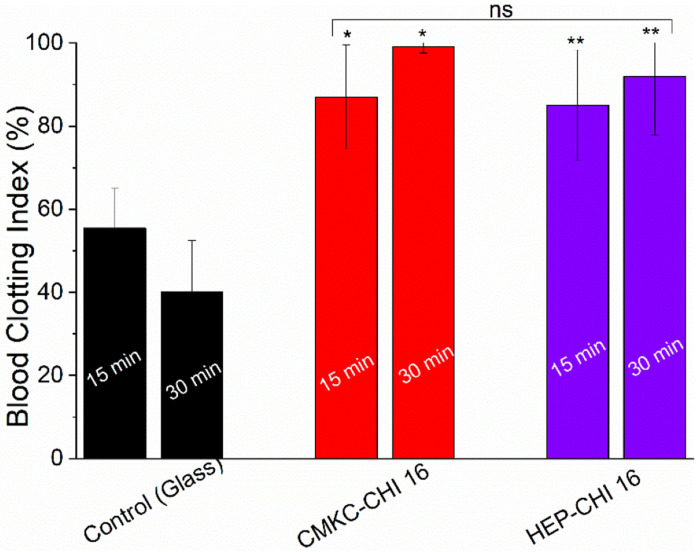
Whole blood clotting measured by the normalized amount of free hemoglobin in human whole blood incubated with PEMs for 15- and 30-min. Reduced blood clotting index indicates increased clotting. * *p* ≤ 0.05 compared to the glass control and “ns” *p* ≥ 0.05 compared between CMKC-CHI 16 and HEP-CHI 16.

Mature blood clots trap red blood cells. After application of whole blood to the samples, the free hemoglobin is released and measured. Red blood cells trapped in the clot do not release their hemoglobin, resulting in a lower blood clot index (BCI) when a clot is more mature. The whole blood clotting assay was used to investigate the coagulation behavior of blood in contact with different biomaterial surfaces. The BCI for control glass, CMKC-CHI, and HEP-CHI PEMs was measured at two time points: 15 and 30 minutes (Figure 9). The BCI provides an estimate of thrombogenic potential, with lower values indicating a higher tendency for clot formation.

The results of the whole blood clotting test show a significant difference in the coagulation characteristics of the modified surfaces when compared to the control. After 15 minutes, the BCI for CMKC-CHI and HEP-CHI surfaces was approximately 55% higher than that of the control glass. This indicates a stronger anticoagulant effect of these surfaces. After 30 minutes, the BCI on the glass continues to fall (indicating continued clotting) whereas the BCI on both CMKC-CHI and HEP-CHI exhibits an increase indicating that any blood clotting is already being reversed (e.g., by activation of plasminogen into plasmin within the clot). This could be linked to the influence of surface modification on protein adsorption and conformation, as well as platelet adhesion and activation. The elevated BCI values demonstrate that the CMKC-CHI PEM effectively prevents blood clot formation, aligning with the preferred characteristics for blood-contacting medical devices. CMKC-CHI surfaces also showed a high BCI, comparable to the HEP-CHI samples, indicating decreased clot formation. The comparison of CMKC-CHI and HEP-CHI surfaces shows no notable difference in their anticoagulant effects. This finding is significant, especially considering the well-documented anticoagulant properties of heparin, a key component of the HEP-CHI modification. The similarity in anticoagulant efficacy between CMKC-CHI and HEP-CHI suggests that CMKC could be a promising alternative to heparin. The lower number of white blood cells on CMKC-CHI surfaces, as compared to HEP-CHI PEM (as illustrated in Figure 7), suggests a reduced likelihood of immune response activation. This characteristic has the potential to improve the biocompatibility of CMKC-CHI and decrease complications related to immune responses in clinical applications. Moreover, the utilization of CMKC, a plant-derived material, aligns with the growing demand for sustainable and ethically sourced biomaterials. In the search for eco-friendly and renewable resources for medical applications, the plant-based nature of CMKC provides significant benefits compared to animal-derived heparin.

## Conclusions

The study findings indicated that the hemocompatibility of blood-contacting surfaces was significantly enhanced by CMKC-CHI polyelectrolyte multilayers. The blood compatibility outcomes include cytocompatibility, antibacterial activity, protein adsorption characteristics, platelet adhesion and activation, and whole blood clotting kinetics. The precise deposition of the CMKC-CHI PEM was confirmed through advanced surface analysis, with notable reductions observed in protein adsorption and platelet activation, while antibacterial properties against *P. aeruginosa* and *S. aureus* were improved. Promising performance of CMKC-terminated PEMs as an alternative to heparin-containing coatings was demonstrated. It was shown that this new approach could potentially enhance the safety and effectiveness of cardiovascular devices by reducing blood clotting and improving biocompatibility.

## Supplementary Material

This is a list of supplementary files associated with this preprint. Click to download.

• SupInfo7.06.25LM.docx

Supplementary Information available: [details of any supplementary information available should be included here]. See DOI: 10.1039/x0xx00000x

## Figures and Tables

**Table 1.** Nitrogen content (on a C, O, N, S basis) of the PEMs before and after protein adsorption experiments, obtained from XPS high-resolution spectra.

PEM		Atom %N
HEP-CHI 16	PEMs	3.83
	PEM + Fib	5.64
	PEM + Alb	10.31
CMKC-CHI 16	PEMs	2.33
	PEM + Fib	9.55
	PEM + Alb	6.74

## Data Availability

Detailed methods for the carboxymethylation of KC, polyelectrolyte multilayer formation, surface characterization, cell culture, cell viability and cytotoxicity assays, cell adhesion and proliferation assays, antibacterial activity assays are all provided in section 1 of the supporting information. XPS results, outcomes form the cell viability and toxicity assays, antibacterial activity assays, protein adsorption assays, and platelet adhesion assays are all provided in section 2 of the supporting information. (pdf) All of the raw data supporting this article are also available as supporting information. (zip)

## References

[1] Arokiarajan M. S., Thirunavukkarasu R., Joseph J., Ekaterina O. and Aruni W., Int J Biol Macromol, 2022, 194, 870–881. 34843816 10.1016/j.ijbiomac.2021.11.142

[2] Avcu E., Bastan F. E., Guney M., Yildiran Avcu Y., Ur Rehman M. A. and Boccaccini A. R., Acta Biomater, 2022, 151, 1–44. 35921991 10.1016/j.actbio.2022.07.048

[3] Madruga L. Y. C., Sabino R. M., Santos E. C. G., Popat K. C., Balaban R. de C. and Kipper M. J., Int J Biol Macromol, 2020, 152, 483–491. 32109473 10.1016/j.ijbiomac.2020.02.274

[4] Bedini E., Laezza A., Parrilli M. and Iadonisi A., Carbohydr Polym, 2017, 174, 1224–1239. 28821048 10.1016/j.carbpol.2017.07.017

[5] Arlov Ø., Rütsche D., Asadi Korayem M., Öztürk E. and Zenobi-Wong M., Adv Funct Mater, 2021, 31, 2010732.

[6] Chess E. K., Bairstow S., Donovan S., Havel K., Hu P., Johnson R. J., Lee S., McKee J., Miller R., Moore E., Nordhaus M., Ray J., Szabo C. and Wielgos T., in Heparin - A Century of Progress, eds. Lever R., Mulloy B. and Page C. P., Springer Berlin Heidelberg, Berlin, Heidelberg, 2012, pp. 99–125. 10.1007/978-3-642-23056-1_6 22566223

[7] Vilanova E., Tovar A. M. F. and Mourão P. A. S., Journal of Thrombosis and Haemostasis, 2019, 17, 254–256. 30582884 10.1111/jth.14372

[8] Daud J. M., Warzukni N. S., Salim R. M. and Zuberdi A. M., Oriental Journal of Chemistry, 2015, 31, 973.

[9] Pacheco-Quito E.-M., Ruiz-Caro R. and Veiga M.-D., Mar Drugs, 2020, 18, 583. 33238488 10.3390/md18110583 PMC7700686

[10] Campo V. L., Kawano D. F., da Silva D. B. and Carvalho I., Carbohydr Polym, 2009, 77, 167–180.

[11] Caputo H. E., Straub J. E. and Grinstaff M. W., Chem Soc Rev, 2019, 48, 2338–2365. 30742140 10.1039/c7cs00593h

[12] Dev A., Mohanbhai S. J., Kushwaha A. C., Sood A., Sardoiwala M. N., Choudhury S. R. and Karmakar S., Acta Biomater, 2020, 109, 121–131. 32335311 10.1016/j.actbio.2020.03.023

[13] Madruga L. Y. C., da Câmara P. C. F., Marques N. do N. and Balaban R. de C., J Mol Liq, 2018, 266, 870–879.

[14] Madruga L. Y. C., Balaban R. C., Popat K. C. and Kipper M. J., Macromol Biosci, 2021, 21, 2000292. 10.1002/mabi.202000292 33021064

[15] Madruga L. Y. C., Popat K. C., Balaban R. C. and Kipper M. J., Carbohydr Polym, 2021, 273, 118541. 34560953 10.1016/j.carbpol.2021.118541

[16] Abbasi-Ravasjani S., Seddiqi H., Moghaddaszadeh A., Ghiasvand M.-E., Jin J., Oliaei E., Bacabac R. G. and Klein-Nulend J., Front Bioeng Biotechnol, 2022, 10, 957263. 36213076 10.3389/fbioe.2022.957263 PMC9542643

[17] Amirtharaj Mosas K. K., Chandrasekar A. R., Dasan A., Pakseresht A. and Galusek D., Gels, 2022, 8, 323. 35621621 10.3390/gels8050323 PMC9140433

[18] Crago M., Lee A., Hoang T. P., Talebian S. and Naficy S., Acta Biomater, 2024, 180, 46–60. 38615811 10.1016/j.actbio.2024.04.018

[19] Wen L., Qiu H., Li S., Huang Y., Tu Q., Lyu N., Mou X., Luo X., Zhou J., Chen Y., Wang C., Huang N. and Xu J., Acta Biomater, 2024, 179, 371–384. 38382829 10.1016/j.actbio.2024.02.022

[20] Sarode A., Annapragada A., Guo J. and Mitragotri S., Biomaterials, 2020, 242, 119929. 32163750 10.1016/j.biomaterials.2020.119929

[21] Criado-Gonzalez M., Mijangos C. and Hernández R., Polymers (Basel), 2021, 13, 2254. 34301010 10.3390/polym13142254 PMC8309355

[22] Baghersad S., Madruga L. Y. C., Martins A. F., Popat K. C. and Kipper M. J., J Funct Biomater, 2023, 14, 554. 37998123 10.3390/jfb14110554 PMC10672460

[23] Escobar A., Muzzio N. and Moya S. E., Pharmaceutics, 2021, 13, 16.

[24] Séon L., Lavalle P., Schaaf P. and Boulmedais F., Langmuir, 2015, 31, 12856–12872. 26513437 10.1021/acs.langmuir.5b02768

[25] Sun G., Li Y., Liu C., Jiang X., Yang L., He L., Song S., Zhang J., Shen J. and Qiao T., ACS Appl Bio Mater, 2022, 5, 2928–2934. 10.1021/acsabm.2c00266 35623056

[26] Li L., Yu Y., Sun X., Wang X., Yang X., Yu Q., Kang K., Wu Y. and Yi Q., J. Mater. Chem. B, 2024, 12, 4184–4196. 38592788 10.1039/d4tb00363b

[27] Li B., Shu Y., Ma H., Cao K., Cheng Y. Y., Jia Z., Ma X., Wang H. and Song K., Tissue Cell, 2024, 87, 102304. 38219450 10.1016/j.tice.2024.102304

[28] Gao H., Hu P., Sun G., Wang L., Tian Y., Mo H., Liu C., Zhang J. and Shen J., J. Mater. Chem. B, 2022, 10, 1077–1084. 35080577 10.1039/d1tb02631c

[29] Vlcek J. R., Madruga L. Y. C., Reynolds M. M. and Kipper M. J., ACS Appl Polym Mater, 2022, 4, 5975–5987.

[30] Vlcek J. R., Reynolds M. M. and Kipper M. J., Biomacromolecules, 2021, 22, 3913–3925. 34347454 10.1021/acs.biomac.1c00720

[31] Almodóvar J., Bacon S., Gogolski J., Kisiday J. D. and Kipper M. J., Biomacromolecules, 2010, 11, 2629–2639. 20795698 10.1021/bm1005799

[32] Sabino R. M., Mondini G., Kipper M. J., Martins A. F. and Popat K. C., Carbohydr Polym, 2021, 251, 117079. 33142622 10.1016/j.carbpol.2020.117079 PMC7717535

[33] Sabino R. M., Kauk K., Madruga L. Y. C., Kipper M. J., Martins A. F. and Popat K. C., J Biomed Mater Res A, 2020, 108, 992–1005. 31909867 10.1002/jbm.a.36876 PMC11448313

[34] Almodóvar J. and Kipper M. J., Macromol Biosci, 2011, 11, 72–76. 20976723 10.1002/mabi.201000261

[35] Almodóvar J., Mower J., Banerjee A., Sarkar A. K., Ehrhart N. P. and Kipper M. J., Biotechnol Bioeng, 2013, 110, 609–618. 22903591 10.1002/bit.24710

[36] Madruga L. Y. C., Sabino R. M., Santos E. C. G., Popat K. C., Balaban R. de C. and Kipper M. J., Int J Biol Macromol, 2020, 152, 483–491. 32109473 10.1016/j.ijbiomac.2020.02.274

[37] Facchi D. P., Lima A. C., de Oliveira J. H., Lazarin-Bidóia D., V Nakamura C., Canesin E. A., Bonafé E. G., Monteiro J. P., V Visentainer J., Muniz E. C. and Martins A. F., Int J Biol Macromol, 2017, 103, 129–138. 28501603 10.1016/j.ijbiomac.2017.05.033

[38] Baghersad S., Bolandi B., Imani R., Afaghi S. and Davoudinia S., J Bionic Eng, 2024, 21, 674–693.

[39] Almodóvar J., Place L. W., Gogolski J., Erickson K. and Kipper M. J., Biomacromolecules, 2011, 12, 2755–2765. 21644518 10.1021/bm200519y

[40] Pahal S., Boranna R., Prashanth G. R. and Varma M. M., Macromol Chem Phys, 2022, 223, 2100330.

[41] Boddohi S., Killingsworth C. E. and Kipper M. J., Biomacromolecules, 2008, 9, 2021–2028. 18564872 10.1021/bm8002573

[42] Nazarzadeh Zare E., Khorsandi D., Zarepour A., Yilmaz H., Agarwal T., Hooshmand S., Mohammadinejad R., Ozdemir F., Sahin O., Adiguzel S., Khan H., Zarrabi A., Sharifi E., Kumar A, Mostafavi E., Kouchehbaghi N. H., Mattoli V., Zhang F., Jucaud V., Najafabadi A. H. and Khademhosseini A., Bioact Mater, 2024, 31, 87–118. 37609108 10.1016/j.bioactmat.2023.08.002 PMC10440395

[43] da Câmara P. C. F., Madruga L. Y. C., Sabino R. M., Vlcek J., Balaban R. C., Popat K. C., Martins A. F. and Kipper M. J., Materials Science and Engineering: C, 2020, 112, 110919. 32409070 10.1016/j.msec.2020.110919

[44] Elsabee M. Z., Morsi R. E. and Al-Sabagh A. M., Colloids Surf B Biointerfaces, 2009, 74, 1–16. 19682870 10.1016/j.colsurfb.2009.06.021

[45] Philippova O. E. and V Korchagina E., Polymer Science Series A, 2012, 54, 552–572.

[46] Tangpasuthadol V., Pongchaisirikul N. and Hoven V. P., Carbohydr Res, 2003, 338, 937–942. 12681917 10.1016/s0008-6215(03)00038-7

[47] Lord M. S., Foss M. and Besenbacher F., Nano Today, 2010, 5, 66–78.

[48] Jin S., Wen J., Zhang Y., Mou P., Luo Z., Cai Y., Chen A., Fu X., Meng W., Zhou Z., Li J. and Zeng W., Acta Biomater, 2024, 177, 91–106. 38311198 10.1016/j.actbio.2024.01.043

[49] Gittens R. A., McLachlan T., Olivares-Navarrete R., Cai Y., Berner S., Tannenbaum R., Schwartz Z., Sandhage K. H. and Boyan B. D., Biomaterials, 2011, 32, 3395–3403. 21310480 10.1016/j.biomaterials.2011.01.029 PMC3350795

[50] Komeri R., Kasoju N. and Anil Kumar P. R., in Biomedical Product and Materials Evaluation, ed. Mohanan P. V, Woodhead Publishing, 2022, pp. 329–353.

[51] Kumar P., Nagarajan A. and Uchil P. D., Cold Spring Harb Protoc, 2018, 2018, pdbprot095497. 10.1101/pdb.top096222 30510131

[52] Martinez J. S., Keller T. C. S. I. I. I. and Schlenoff J. B., Biomacromolecules, 2011, 12, 4063–4070. 22026411 10.1021/bm201142x PMC3216489

[53] Niepel M. S., Ekambaram B. K., Schmelzer C. E. H. and Groth T., Nanoscale, 2019, 11, 2878–2891. 30688341 10.1039/c8nr05529g

[54] Sun D., Babar Shahzad M., Li M., Wang G. and Xu D., Materials Technology, 2015, 30, B90–B95.

[55] Wang L., Hu C. and Shao L., Int J Nanomedicine, 2017, 12, 1227–1249. 28243086 10.2147/IJN.S121956 PMC5317269

[56] Pajerski W., Ochonska D., Brzychczy-Wloch M., Indyka P., Jarosz M., Golda-Cepa M., Sojka Z. and Kotarba A., Journal of Nanoparticle Research, 2019, 21, 186.

[57] Akbari A., Sadani M., Amin M. M., Teimouri F., Khajeh M., Mahdavi M. and Hadi M., J Environ Chem Eng, 2018, 6, 5929–5937.

[58] Salton M. R. J., Biochim Biophys Acta, 1953, 10, 512–523. 13059016 10.1016/0006-3002(53)90296-0

[59] Swain J., El Khoury M., Flament A., Dezanet C., Briée F., Van Der Smissen P., Décout J.-L. and Mingeot-Leclercq M.-P., Biochimica et Biophysica Acta (BBA) - Biomembranes, 2019, 1861, 182998. 31153908 10.1016/j.bbamem.2019.05.020

[60] Zhang G., Meredith T. C. and Kahne D., Curr Opin Microbiol, 2013, 16, 779–785. 24148302 10.1016/j.mib.2013.09.007 PMC3974409

[61] Caroff M. and Novikov A., OCL, 2020, 27, 31.

[62] Sperling C., Fischer M., Maitz M. F. and Werner C., Biomaterials, 2009, 30, 4447–4456. 19535136 10.1016/j.biomaterials.2009.05.044

[63] Yamazoe H., Oyane A., Nashima T. and Ito A., Materials Science and Engineering: C, 2010, 30, 812–816.

[64] Sivaraman B. and Latour R. A., Biomaterials, 2010, 31, 832–839. 19850334 10.1016/j.biomaterials.2009.10.008 PMC2790000

[65] Xu L.-C., Bauer J. W. and Siedlecki C. A., Colloids Surf B Biointerfaces, 2014, 124, 49–68. 25448722 10.1016/j.colsurfb.2014.09.040 PMC5001692

[66] Brass L. F., Jiang H., Wu J., Stalker T. J. and Zhu L., Blood Cells Mol Dis, 2006, 36, 157–161. 16473534 10.1016/j.bcmd.2005.12.015

[67] Grunkemeier J. M., Tsai W. B., McFarland C. D. and Horbett T. A., Biomaterials, 2000, 21, 2243–2252. 11026630 10.1016/s0142-9612(00)00150-2

[68] Ben-Ami R., Barshtein G., Mardi T., Deutch V., Elkayam O., Yedgar S. and Berliner S., Am J Physiol Heart Circ Physiol, 2003, 285, H2663–H2669. 12869382 10.1152/ajpheart.00128.2003

[69] Köhler S., Schmid F. and Settanni G., PLoS Comput Biol, 2015, 11, e1004346. 26366880 10.1371/journal.pcbi.1004346 PMC4569070

[70] Protopopova A. D., Barinov N. A., Zavyalova E. G., Kopylov A. M., Sergienko V. I. and Klinov D. V., Journal of Thrombosis and Haemostasis, 2015, 13, 570–579. 25393591 10.1111/jth.12785

[71] Soman P., Rice Z. and Siedlecki C. A., Langmuir, 2008, 24, 8801–8806. 18616311 10.1021/la801227e

[72] Simon-Walker R., Romero R., Staver J. M., Zang Y., Reynolds M. M., Popat K. C. and Kipper M. J., ACS Biomater Sci Eng, 2017, 3, 68–77. 33429688 10.1021/acsbiomaterials.6b00572

[73] Ko T. and Cooper S. L., J Appl Polym Sci, 1993, 47, 1601–1619.

[74] Goodman S. L., Grasel T. G., Cooper S. L. and Albrecht R. M., J Biomed Mater Res, 1989, 23, 105–123. 10.1002/jbm.820230109 2708401

[75] Hedayati M., Kipper M. J. and Krapf D., Physical Chemistry Chemical Physics, 2020, 22, 5264–5271. 32095800 10.1039/d0cp00326c

[76] Hedayati M., Marruecos D. F., Krapf D., Kaar J. L. and Kipper M. J., Acta Biomater, 2020, 102, 169–180. 31731023 10.1016/j.actbio.2019.11.019

[77] Hedayati M., Neufeld M. J., Reynolds M. M. and Kipper M. J., Elsevier Ltd, 2019, preprint, DOI: 10.1016/j.mser.2019.06.002.

